# Evaluation of Compatibility of Different Attachment Types Used in Orthodontic Clear Aligners with Electron Microscopy

**DOI:** 10.3390/dj13080379

**Published:** 2025-08-20

**Authors:** Can Sever, Can Arslan

**Affiliations:** Department of Orthodontics, Faculty of Dentistry, Yeditepe University, Istanbul 34728, Turkey; can.arslan@yeditepe.edu.tr

**Keywords:** clear aligners, orthodontic attachments, scanning electron microscopy, material adaptation, thermoforming

## Abstract

**Background/Objectives**: The effectiveness of clear aligner therapy depends significantly on the precision of force delivery through the aligner–attachment interface. This study aimed to evaluate the microscopic compatibility between different orthodontic clear aligner materials (Duran+ and Zendura FLX) and attachment designs (rectangular and optimized) using scanning electron microscopy (SEM). **Methods**: Fifty-six samples were divided into four groups: rectangular attachments with Duran+ aligners (n = 14), rectangular attachments with Zendura FLX aligners (n = 14), optimized attachments with Duran+ aligners (n = 14), and optimized attachments with Zendura FLX aligners (n = 14). Attachments were bonded to bovine incisors using standardized protocols. Clear aligners were thermoformed at 220 °C for 40 s. Cross-sectional samples were analyzed using SEM at 250× magnification. Gap measurements were taken at seven points for rectangular attachments and five points for optimized attachments. **Results**: Gap measurements ranged from 14.75 ± 1.41 µm to 91.07 ± 3.11 µm. Zendura FLX demonstrated significantly better adaptation than Duran+ with rectangular attachments (42.10 ± 1.07 µm vs. 44.52 ± 1.51 µm, *p* < 0.001). Optimized attachments showed better overall adaptation than rectangular attachments. All combinations showed regional variation with the largest gaps at gingival borders (67.18–91.07 µm) and the smallest at flat buccal surfaces (14.75–20.98 µm). **Conclusions**: Perfect adaptation was not achieved with any material–attachment combination tested. Material selection and attachment design significantly influence microscopic adaptation, with multi-layer materials and optimized geometries showing superior performance. These findings provide mechanical explanations for clinical limitations in clear aligner therapy.

## 1. Introduction

The increasing demand for aesthetic orthodontic treatment has led to widespread adoption of clear aligner therapy [1,2]. Since their introduction by Kesling in the 1940s, clear aligners have evolved from simple retention devices to sophisticated orthodontic appliances capable of complex tooth movements [3,4]. This evolution has been facilitated by advances in material science, digital technology, and the introduction of attachments—composite resin structures bonded to teeth to enhance force delivery and control tooth movement [5,6].

Clear aligner treatment has become increasingly popular due to advantages including aesthetics, patient comfort, and improved oral hygiene compared to fixed appliances [7,8]. The development of computer-aided design and manufacturing (CAD/CAM) technology has revolutionized aligner production [9,10]. Recent innovations have transformed clear aligner therapy through multiple technological advances. Artificial intelligence applications now extend beyond manufacturing to comprehensive treatment planning, with AI-powered systems helping assess facial aesthetics and determine optimal treatment objectives for improved patient outcomes [11]. Direct 3D printing technologies have emerged as an alternative to traditional thermoforming, potentially eliminating manufacturing limitations [12]. These technological advances, combined with novel multi-layer aligner materials, have enhanced both the precision of treatment planning and the mechanical properties of aligners [13]. Modern aligner systems incorporate various attachment designs to facilitate complex movements previously thought impossible with removable appliances [14]. Recent biomechanical studies have quantified improvements in force delivery systems, with attachment designs demonstrating enhanced force control [15,16] and multi-layer materials showing superior mechanical properties [13], collectively enabling more predictable orthodontic tooth movement.

Despite technological advances, achieving predictable tooth movement with clear aligners remains challenging. Clinical studies report varying success rates for different movements, with certain movements such as extrusion, rotation, and root control showing limited predictability [17,18,19]. Djeu et al. found that while aligners achieved comparable results to fixed appliances for some parameters, they showed limitations in correcting buccolingual inclination, occlusal contacts, and occlusal relationships [20]. These clinical limitations suggest underlying mechanical factors that warrant investigation.

The effectiveness of clear aligner therapy depends significantly on the precision of force delivery through the aligner–attachment interface [15,16]. Previous research has demonstrated that attachment design influences force systems and movement predictability [21,22]. However, the microscopic relationship between aligners and attachments, which directly affects force transmission efficiency, remains incompletely understood. Various measurement techniques including tensile testing, silicone replication methods, and optical scanning have been employed to evaluate aligner fit, each with inherent limitations [6,23,24].

Recent studies using advanced imaging techniques have begun to reveal the complexity of the aligner–attachment interface. Contemporary research has focused on optimizing this interface through novel materials and manufacturing techniques [25]. Mantovani et al. pioneered the use of scanning electron microscopy to evaluate aligner fit, demonstrating measurable gaps between aligners and dental structures [26,27]. Lombardo et al. used micro-computed tomography to compare different aligner brands, finding significant variations in gap dimensions [28]. The measurable gaps identified by Mantovani et al. and the brand-specific variations reported by Lombardo et al. suggest that microscopic adaptation may be a critical factor influencing clinical outcomes. The clinical significance of gap dimensions has been investigated by multiple researchers. Barone et al. [29] identified 50 μm as a critical threshold for compromised force transmission. Supporting this, Elkholy et al. [30] found 40% force reduction with gaps exceeding 100 μm, while Hahn et al. [31] reported clinically significant gaps ranging from 50 to 200 μm. These convergent findings emphasize the importance of quantifying microscopic adaptation.

This study aimed to quantitatively assess microscopic adaptation at the aligner–attachment interface by comparing two aligner materials (Duran+ and Zendura FLX) and two attachment designs (rectangular and optimized) using scanning electron microscopy. The null hypothesis was that perfect adaptation (100% fit) exists between aligners and attachments regardless of material type or attachment design.

## 2. Materials and Methods

### 2.1. Study Design and Sample Size

This in vitro study was designed to compare the microscopic adaptation between two aligner materials and two attachment designs. Power analysis using G*Power 3.1 software determined a sample size of 56 (14 per group) to achieve 80% power with α = 0.05, based on an effect size of 0.46 for gap measurements between aligners and attachments [32].

### 2.2. Specimen Preparation

Fifty-six caries-free bovine mandibular incisors were selected based on standardized criteria: intact enamel surface, no structural anomalies, and similar crown dimensions. The use of bovine teeth follows established protocols in dental research due to their availability and comparable bonding characteristics to human enamel [33,34]. Teeth were cleaned with pumice and stored in distilled water at room temperature. Each tooth was mounted in cold-cure acrylic resin and scanned using a 3D scanner (3Shape E1 Series, Copenhagen, Denmark) with accuracy within 7–10 µm [35]. All specimen preparation steps, including tooth mounting, attachment bonding, aligner fabrication, and sectioning, were performed by a single trained investigator (C.S.) to ensure consistency and eliminate inter-operator variability.

### 2.3. Attachment Design and Production

Two attachment designs were created using Autodesk MeshMixer (Version 3.5.474, San Rafael, CA, USA):

- Rectangular attachment: 3 mm width × 5 mm length × 2 mm height;
- Optimized attachment: 3 mm width × 5 mm length × 2.5 mm height with curved surfaces.

These dimensions were selected based on commonly used clinical parameters and previous research [6,21]. Attachment templates were designed with 0.1 mm offset following established protocols [36] and 2 mm thickness based on material testing data [37]. Templates were 3D printed using a Form 3 printer (Formlabs, Somerville, MA, USA) with IBT resin at 0.1 mm layer thickness. The IBT resin used for template fabrication provided sufficient flexibility (flexural modulus: 2.2 GPa) to adapt to convex tooth surfaces while maintaining dimensional stability during composite placement. The template design was specifically modified with extended margins and flexible borders to accommodate the natural convexity of bovine incisors. The 2 mm template thickness was specifically chosen to balance flexibility for adaptation with rigidity for accurate attachment positioning. Prior to clinical use, each template was tested on the corresponding tooth model to ensure complete marginal adaptation without gaps. Post-processing included washing in 99% isopropyl alcohol for 20 min and UV curing at 60 °C for 60 min [38].

**Figure 1:**
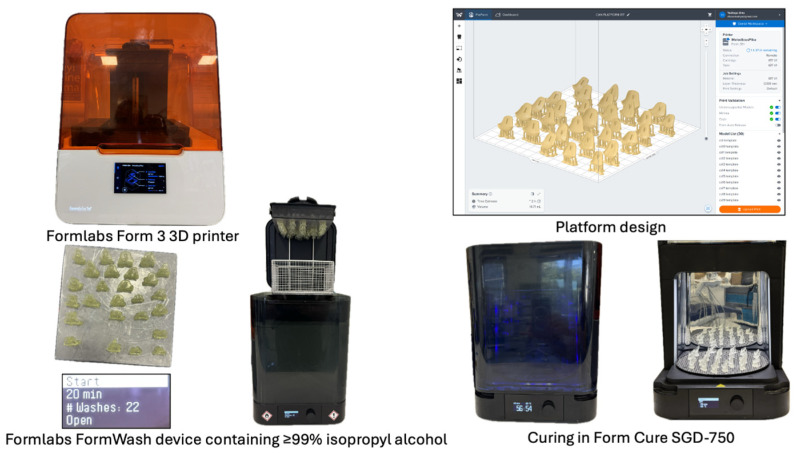
Attachment template production chart.

The complete workflow from digital attachment design to template production is illustrated in Figure 1, demonstrating the step-by-step process for creating standardized attachment templates.

### 2.4. Attachment Bonding Protocol

Standardized bonding protocol was followed for all specimens:

1. Enamel etching with 37% phosphoric acid for 30 s [39];
2. Application of bonding agent (Transbond XT Primer, 3M Unitek, Monrovia, CA, USA) [40];
3. Light curing for 20 s using Woodpecker Dental iLed (Guilin, China);
4. Composite placement (Transbond XT, 3M Unitek) in template;
5. Template positioning with 50 g standardized force measured with Dentaurum force gauge;
6. Light curing for 40 s [41];
7. Following template removal, excess composite material was carefully removed using a scalpel blade under magnification, ensuring attachment margins were smooth without altering the designed dimensions.

Attachments were positioned at the buccal surface center, 4 mm from the incisal edge, using a digital guide. A custom jig ensured all specimens were sectioned at identical angles through the attachment center.

### 2.5. Aligner Fabrication

Two aligner materials were tested:

- Duran+ (Scheu Dental, Iserlohn, Germany): 0.76 mm single-layer PETG;
- Zendura FLX (Bay Materials, Fremont, CA, USA): 0.76 mm multi-layer polyurethane.

Duran+ is a single-layer PETG material (flexural modulus: 2200 MPa), while Zendura FLX features a three-layer construction with an elastomeric core between rigid outer layers (combined flexural modulus: 1100 MPa), providing enhanced flexibility.

Material selection was based on their widespread clinical use and different mechanical properties [42,43]. Aligners were thermoformed individually over each mounted tooth specimen to ensure standardization and prevent processing variables that could occur with group thermoforming. Aligners were thermoformed using a Biostar device (Scheu Dental) at 220 °C for 40 s with ≥4 bar pressure, following manufacturer recommendations [44]. No spacer foils were used to evaluate direct material adaptation. Aligners were trimmed 2 mm beyond the gingival margin using standardized protocols [45].

### 2.6. Scanning Electron Microscopy Analysis

**Figure 2:**
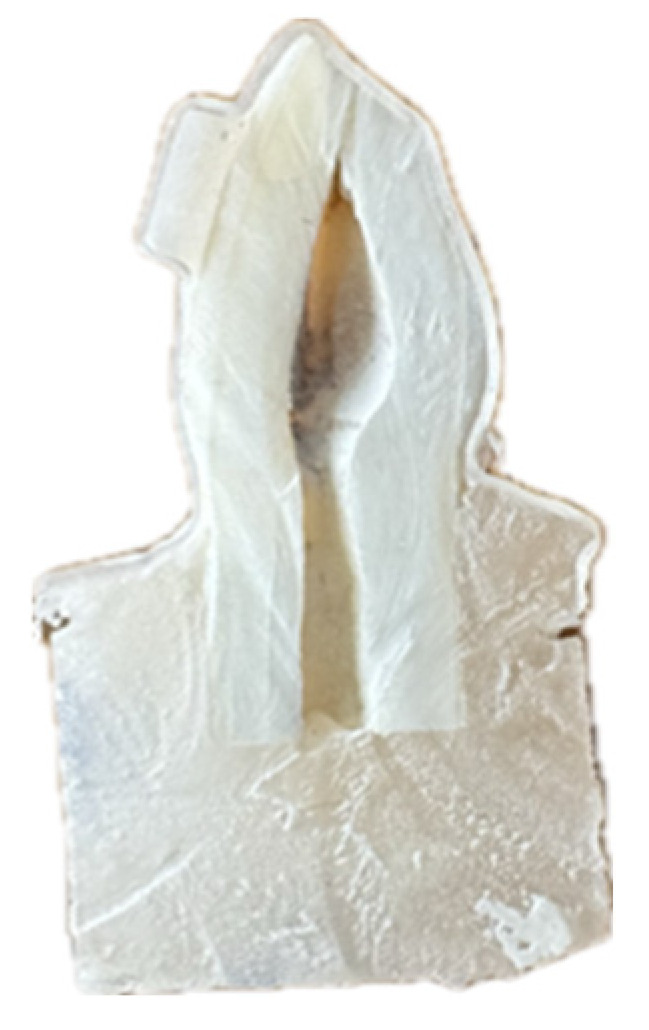
Cross-sectioned tooth sample.

Samples were sectioned buccolingually using a precision cutting machine (Saeshin Strong 210, Daegu, Republic of Korea) with a diamond-embedded disc (Acurata GmbH, Thurmansbang, Germany) under water cooling to prevent thermal deformation [46]. To minimize mechanical deformation during sectioning, samples were embedded in cold-cure epoxy resin prior to cutting, providing support to maintain aligner–tooth interface integrity. Cutting speed was maintained at 500 rpm with minimal pressure to avoid compression artifacts. Post-sectioning examination under light microscopy confirmed the absence of visible deformation or separation at the aligner–attachment interface before SEM analysis. Figure 2 shows a representative cross-sectioned tooth sample prepared for SEM analysis.

**Figure 3:**
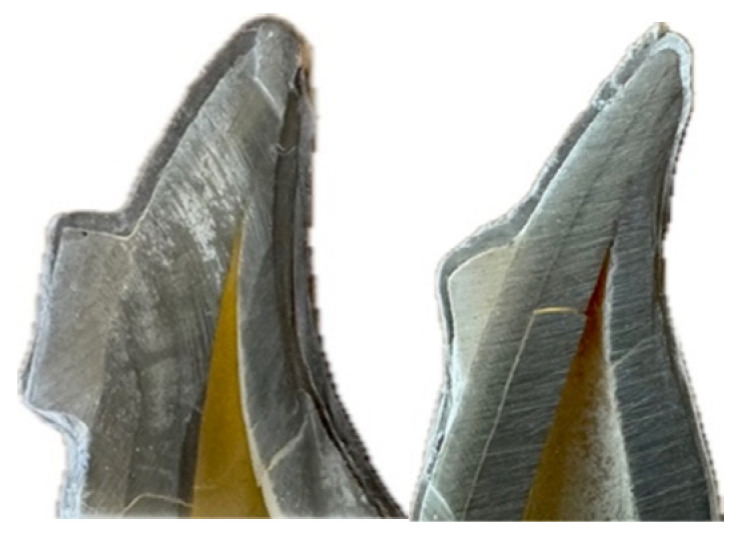
Coating tooth samples.

Cross-sections were coated with gold-palladium using a Leica EM ACE600 (Wetzlar, Germany) sputter coater to enhance imaging quality [47,48] (Figure 3).

Samples were examined using EVO 40 Series SEM (Carl Zeiss AG, Jena, Germany) at 15 kV acceleration voltage, 10 mm working distance, and 250× magnification [49].

**Figure 4:**
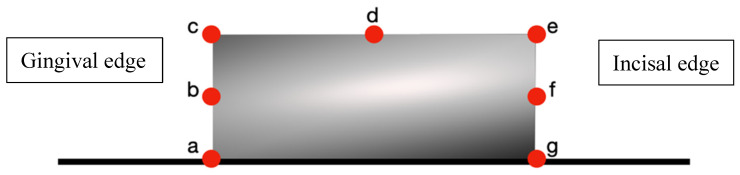
Seven points measured on the rectangular attachment.

Gap measurements were performed at standardized points:

- Rectangular attachments: 7 measurement points (Figure 4);

Figure 4 measurement points on rectangular attachments: (a) gingival border—aligner–tooth junction at gingival margin, (b) gingival midpoint—center of gingival attachment surface, (c) gingival angle—transition between gingival and buccal surfaces, (d) buccal midpoint—center of buccal attachment surface, (e) occlusal angle—transition between buccal and occlusal surfaces, (f) occlusal midpoint—center of occlusal attachment surface, (g) occlusal border—aligner–tooth junction at occlusal margin.

**Figure 5:**
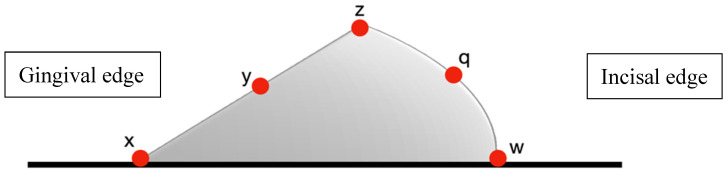
Five points measured on the optimized attachment.

- Optimized attachments: 5 measurement points (Figure 5);

Figure 5 measurement points on optimized attachments: (x) gingival border—aligner–tooth junction at the gingival margin where the maximum gap typically occurs, (y) gingival midpoint—center of the curved gingival surface assessing adaptation to beveled design, (z) buccal angle—critical transition point between the attachment apex and tooth surface, (q) occlusal midpoint—center of the gradual occlusal slope, (w) occlusal border—aligner–tooth junction at the occlusal margin. The reduced number of measurement points (5 vs. 7) reflects the smoother contours and absence of sharp angles in optimized attachment geometry.

Measurement points were strategically selected to account for known regional variations in thermoformed aligner adaptation, encompassing gingival, middle, and occlusal thirds of the clinical crown.

Measurement points were systematically selected to represent the following: (1) areas of maximum stress concentration based on attachment geometry, (2) transition zones between the attachment and tooth surface, and (3) standardized locations across gingival-occlusal dimension for inter-group comparison. The rectangular attachment required seven points to adequately assess all geometric discontinuities, while the optimized attachment’s gradual contours were sufficiently characterized with five strategic points.

**Figure 6:**
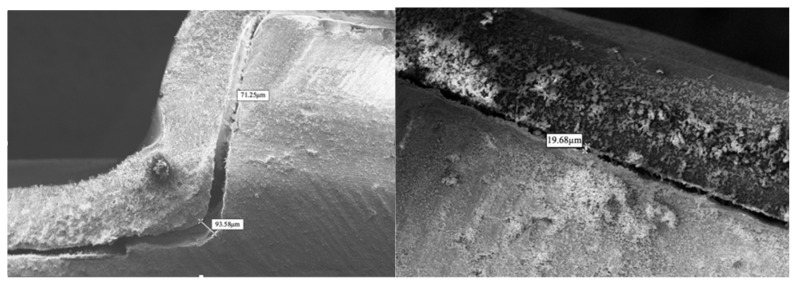
SEM images measurements (Mag = 250×).

All measurements were performed by a single calibrated examiner blinded to group allocation. Figure 6 illustrates representative SEM images showing measurement methodology at 250× magnification.

A single-examiner design was chosen following consultation with biostatistics experts, as the primary outcome (gap measurement) involved objective digital measurements using SmartSEM version 6.0 (Carl Zeiss AG) rather than subjective assessment. The examiner’s role was limited to identifying pre-determined anatomical landmarks and initiating software-based measurements. To ensure reliability, strict standardization protocols were implemented including the following: (1) detailed photographic guides for each measurement point, (2) automated measurement tools in SEM software to eliminate manual measurement variability, and (3) excellent intra-examiner reliability testing (ICC > 0.95). Additionally, the examiner was blinded to group allocation to prevent bias. SEM images were randomly coded by an independent researcher, with the coding key sealed until analysis completion. The examiner received randomized, unlabeled images and recorded measurements using only coded identifiers. Group allocation was revealed after all measurements and analyses were completed.

### 2.7. Statistical Analysis

Data were analyzed using SPSS version 24.0 (IBM Corp., Armonk, NY, USA). Normality was assessed using the Shapiro–Wilk test. As data showed non-normal distribution, the Mann–Whitney U test was used for comparisons between groups. Results were expressed as mean ± standard deviation. Significance level was set at *p* < 0.05.

## 3. Results

Gap measurements varied significantly based on measurement location, attachment design, and aligner material. No perfect adaptation was observed in any group, with all samples showing measurable gaps at all measurement points.

### 3.1. Material Comparison

For rectangular attachments, Zendura FLX showed significantly better adaptation than Duran+ at multiple measurement points (Table 1). The average gap distance was 42.10 ± 1.07 µm for Zendura FLX versus 44.52 ± 1.51 µm for Duran+ (*p* < 0.001). Statistically significant differences were observed at the gingival border (*p* = 0.001), gingival midpoint (*p* = 0.022), gingival angle (*p* = 0.023), and occlusal border (*p* = 0.006).

For optimized attachments, differences between materials were less pronounced, with only the buccal angle showing significant difference (Table 2). The average gap distance showed no statistically significant difference between materials (37.30 ± 3.09 µm for Zendura FLX vs. 39.41 ± 3.20 µm for Duran+, *p* = 0.089).

### 3.2. Attachment Design Comparison

Optimized attachments demonstrated better overall adaptation compared to rectangular attachments regardless of aligner material (Table 3). For Duran+ aligners, average gap distances were 39.41 ± 3.20 µm with optimized attachments versus 44.52 ± 1.51 µm with rectangular attachments. For Zendura FLX aligners, average gaps were 37.30 ± 3.09 µm with optimized attachments versus 42.10 ± 1.07 µm with rectangular attachments.

### 3.3. Regional Variation Patterns

Consistent patterns emerged across all groups regarding gap distribution. When analyzing data without considering material type, rectangular attachments showed distinctive regional variations (Table 4).

The pattern of regional variation was as follows:

- Largest gaps consistently occurred at gingival borders (67.18–91.07 µm);
- Intermediate gaps were observed at occlusal borders (38.41–47.28 µm);
- Smallest gaps were found at flat surfaces and acute angles (14.75–20.98 µm).

## 4. Discussion

This study provides quantitative evidence that perfect adaptation between clear aligners and attachments is not achieved with current materials and manufacturing methods. The finding that all tested combinations showed measurable gaps ranging from 14.75 µm to 91.07 µm has important implications for understanding force transmission efficiency and clinical limitations in clear aligner therapy.

### 4.1. Material Properties and Adaptation

The superior adaptation demonstrated by Zendura FLX compared to Duran+ aligns with previous research on material properties affecting aligner performance [50,51]. The multi-layer construction of Zendura FLX, featuring an elastomeric core between rigid outer layers, appears to enhance conformability to complex geometries. This finding is consistent with Cowley et al. who reported that multi-layer materials showed approximately 8–12% better retention than single-layer materials [45].

The 5.5% improvement in average adaptation with Zendura FLX for rectangular attachments suggests that material selection can meaningfully impact force delivery efficiency.

The material differences were more pronounced with rectangular attachments than with optimized attachments, suggesting an interaction between material properties and attachment geometry. This may be explained by the sharp angles of rectangular attachments presenting greater challenges for material adaptation compared to the gradual contours of optimized attachments. Ryokawa et al. demonstrated that material composition significantly influences stress, relaxation, and adaptability to dental surfaces [50], supporting our findings of material-dependent adaptation patterns.

### 4.2. Attachment Design Implications

Optimized attachments showed approximately 11.5% better adaptation compared to rectangular attachments. While our study evaluated geometric adaptation focusing on gradual contours, optimized attachments incorporate additional design principles including force vector optimization, variable thickness profiles, movement-specific orientations, and strategic undercuts for retention. The superior adaptation we observed likely results from both the smoother surface transitions facilitating material flow during thermoforming and the biomechanically optimized shapes creating favorable stress distribution patterns. This multifaceted design approach represents a significant advancement beyond simple geometric shapes, extending the work of Savignano et al. who demonstrated that attachment geometry significantly influences force systems [21].

It is important to note that our study evaluated microscopic gap formation at the aligner–attachment interface, not retention forces. These parameters, while both clinically relevant, measure fundamentally different aspects of aligner performance. Interestingly, our results contrast with those of Dasy et al. who reported superior retention with rectangular attachments [6]. This discrepancy highlights an important distinction: retention tests measure force required to dislodge aligners, while adaptation assessment evaluates contact intimacy for force transmission. Both are clinically significant—retention prevents aligner dislodgement while adaptation ensures efficient force delivery. Poor retention interrupts treatment, but poor adaptation causes unpredictable tooth movement. The superior adaptation of optimized attachments may compensate for potentially lower retention through more efficient force transmission, possibly explaining their clinical preference despite contrasting laboratory retention data.

While our study demonstrated superior adaptation with optimized attachments, it is important to acknowledge their disadvantages documented in recent literature. Optimized attachments show no clear superiority over conventional attachments for many movements, with both types achieving only partial planned movement and often requiring overcorrection in treatment planning [52]. Furthermore, optimized root control attachments demonstrate reduced efficiency due to surface wear, particularly after four months of use, showing greater wear-related performance degradation compared to rectangular attachments [53]. This wear can significantly impact long-term treatment effectiveness, especially for canine distalization, potentially requiring attachment rebonding or restoration during treatment.

Current literature reveals that attachment efficiency is highly movement-specific, with no universal superiority of any single design. For lateral incisor rotation, optimized attachments demonstrate superior performance [54]. However, conventional vertical attachments prove more effective for mesio-distal angulation, while horizontal attachments excel in vestibulo-lingual inclination (torque) movements [54]. For maxillary lateral incisor extrusion between 0.3 and 2.5 mm, horizontal attachments significantly outperform optimized attachments [55]. Rectangular attachments maintain better long-term efficiency for canine distalization due to reduced impact from surface wear [53]. These findings emphasize that attachment selection should be individualized based on the specific tooth movement required rather than defaulting to any single attachment type, with clinicians considering both immediate effectiveness and long-term performance stability.

### 4.3. Regional Variation and Clinical Significance

Our study design specifically addressed the known variation in thermoformed aligner fit across different regions of clinical crowns. By establishing standardized measurement points at gingival, middle, and occlusal regions, we captured the full spectrum of adaptation patterns. This regional assessment approach aligns with previous observations by Krey et al. [43] who demonstrated non-uniform thickness distribution in thermoformed aligners, with up to 50% thickness reduction at gingival margins. Our findings of larger gaps at gingival borders (67.18–91.07 μm) compared to occlusal regions (38.41–47.28 μm) confirm this regional variation pattern and provide quantitative data on its magnitude.

The consistent regional variation pattern observed across all groups provides mechanical insight into clinical observations of movement-specific limitations. The largest gaps at gingival margins (up to 91.07 µm) may explain the limited predictability of extrusive movements reported by Rossini et al. [17], as these movements rely heavily on force application at gingival regions. The smallest gaps observed at flat buccal surfaces may contribute to the efficiency of movements that rely on direct aligner–tooth contact at these regions. However, it is important to note that simple tipping movements often do not require attachments at all, as the aligner itself can provide sufficient force application above the center of resistance [56]. Our findings of superior adaptation at buccal surfaces are more relevant for complex movements requiring precise force control, such as bodily translation or root torque, where attachments become essential for proper force coupling [29]. The regional adaptation patterns we identified should not be interpreted as suggesting attachments are necessary for all movements, but rather as explaining why certain attachment-dependent movements may be more or less predictable based on the quality of the aligner–attachment interface.

Our gap measurements exceed the 50 µm threshold identified by Barone et al. as potentially compromising force transmission efficiency [57]. This finding suggests that current manufacturing methods may inherently limit the precision of certain tooth movements. The regional variation pattern appears to result from thermoforming mechanics, where material stretching and thinning occur non-uniformly. Krey et al. demonstrated that thermoformed aligners can lose up to 50% of their original thickness at gingival margins [43], which may explain the larger gaps observed in these regions. Our regional variation findings align with the well-documented phenomenon of non-uniform thickness distribution in thermoformed aligners. Ryu et al. [58] demonstrated thickness reductions of up to 70% at incisal edges and 50% at gingival margins compared to the original sheet thickness. Similarly, Lombardo et al. [59] reported significant thinning at cusp tips and marginal areas, with thickness variations directly correlating with gap formation. This thickness reduction pattern, most pronounced at aligner borders, provides a mechanical explanation for our finding of largest gaps at gingival margins (67.18–91.07 µm). The thermoforming process inherently creates these variations through differential material stretching, with maximum thinning occurring at areas of greatest draw depth.

### 4.4. Comparison with Previous Studies

Our findings align with and extend previous microscopy studies of aligner adaptation. Mantovani et al. reported average gaps ranging from 22.7 to 80.1 µm depending on measurement location [26], which encompasses our range of measurements. However, our study found a wider overall range (14.75–91.07 µm), possibly due to the inclusion of different attachment designs and systematic evaluation of multiple standardized points.

Lombardo et al. used micro-CT analysis to evaluate six aligner brands and reported mean gaps ranging from 24.3 to 48.9 µm [28]. Their results are consistent with our average measurements but lower than our maximum values. This difference may reflect methodological variations—micro-CT provides volumetric assessment while SEM offers superior resolution for precise point measurements. Each method has strengths, with SEM providing the precision necessary to detect subtle differences in adaptation patterns.

### 4.5. Clinical Implications

These findings have direct implications for clinical practice and treatment planning. The regional adaptation patterns suggest that clinicians should consider location-specific force transmission efficiency when planning tooth movements. Movements primarily engaging regions of better adaptation (flat surfaces) could be planned more aggressively, while movements dependent on regions of poorer adaptation (gingival margins, sharp angles) may benefit from the following:

- More conservative staging with smaller incremental movements;
- Additional attachments to distribute forces across multiple contact points;
- Auxiliary mechanics such as elastics or temporary anchorage devices;
- Selection of multi-layer aligner materials for complex movements;
- Preference for optimized attachment designs when available.

The finding that even the best material–attachment combinations showed significant gaps challenges assumptions about force delivery in clear aligner therapy. This may partially explain why certain movements remain unpredictable despite advances in treatment planning software and attachment design [19,60], even with the latest AI-driven planning systems and biomechanically optimized attachments [61].

### 4.6. Future Directions

The consistent pattern of regional adaptation variation suggests opportunities for technological advancement. Current thermoforming processes appear to have inherent limitations in achieving uniform adaptation across complex geometries. Future innovations might include the following:

- Multi-stage thermoforming with differential pressure application;
- Direct 3D printing of aligners to eliminate thermoforming limitations [38];
- Development of shape-memory materials with improved adaptation properties;
- Hybrid manufacturing combining thermoforming with selective reinforcement;
- Attachment designs specifically optimized for regional adaptation patterns.

### 4.7. Study Limitations

Several limitations should be acknowledged. The use of bovine teeth, while providing standardization, may not fully replicate human enamel characteristics [33]. The static evaluation does not capture dynamic changes during clinical use, including effects of intraoral forces, temperature fluctuations, and material fatigue. The two-dimensional nature of SEM analysis provides detailed information at specific sections but cannot capture three-dimensional variation around attachment perimeters.

Future studies should investigate adaptation under simulated clinical conditions and correlate microscopic findings with treatment outcomes.

## 5. Conclusions

This study demonstrates that perfect adaptation between clear aligners and attachments is not achieved with current materials and methods. Significant differences exist between materials and attachment designs, with multi-layer aligner materials and optimized attachments showing superior microscopic adaptation. Regional variation patterns, with the largest gaps at gingival margins and the smallest at flat surfaces, provide partial mechanical insights into movement-specific limitations in clear aligner therapy, though we acknowledge that clinical outcomes are influenced by multiple factors including patient compliance, biomechanical complexity, treatment planning accuracy, and individual biological responses. These findings support continued development of materials and attachment designs to enhance force transmission efficiency and clinical predictability. Clinicians should consider these adaptation patterns alongside other clinical factors when planning treatment, selecting materials, and setting realistic expectations for tooth movement with clear aligners.

## Figures and Tables

**Table 1:** Comparison of gap measurements (µm) between Duran+ and Zendura FLX aligners in rectangular attachment (mean ± SD).

Measurement Points.	Duran+ (n = 14)	Zendura FLX (n = 14)	*p*-Value
a (Gingival border)	91.07 ± 3.11	85.77 ± 4.46	0.001 **
b (Gingival midpoint)	70.71 ± 2.42	68.51 ± 2.34	0.022 *
c (Gingival angle)	50.78 ± 2.26	48.75 ± 2.16	0.023 *
d (Buccal midpoint)	20.98 ± 2.24	19.88 ± 1.74	0.159
e (Occlusal angle)	15.62 ± 1.58	14.75 ± 1.41	0.138
f (Occlusal midpoint)	18.87 ± 2.611	17.62 ± 2.49	0.206
g (Occlusal border)	43.70 ± 4.123	38.41 ± 3.40	0.006 *

* *p* < 0.05, ** *p* < 0.001.

**Table 2:** Comparison of gap measurements (µm) between Duran+ and Zendura FLX aligners in optimized attachment (mean ± SD).

Measurement Points	Duran+ (n = 14)	Zendura FLX (n = 14)	*p*-Value
x (Gingival border)	69.91 ± 4.87	67.18 ± 4.92	0.152
y (Gingival midpoint)	29.62 ± 3.19	27.68 ± 3.27	0.127
z (Buccal angle)	32.38 ± 3.11	29.70 ± 3.07	0.030 *
q (Occlusal midpoint)	18.25 ± 2.831	16.50 ± 2.96	0.122
w (Occlusal border)	47.28 ± 4.07	45.50 ± 3.93	0.248

* *p* < 0.05.

**Table 3:** Comparison of Average Space Distance (mean ± SD).

Attachment Type	Duran+	Zendura FLX	*p*-Value
Rectangular attachment	44.52 ± 1.51	42.10 ± 1.07	0.000 **
Optimized attachment	39.41 ± 3.20	37.30 ± 3.09	0.089

** *p* < 0.001.

**Table 4:** Gap measurements without considering material classification in rectangular attachment (n = 28).

Measurement Points	Mean ± SD (µm)
a (Gingival border)	88.39 ± 4.62
b (Gingival midpoint)	69.61 ± 2.59
c (Gingival angle)	49.77 ± 2.40
d (Buccal midpoint)	20.43 ± 2.05
e (Occlusal angle)	15.19 ± 1.54
f (Occlusal midpoint)	18.24 ± 2.58
g (Occlusal border)	41.56 ± 4.30

## Data Availability

The raw data supporting the conclusions of this article will be made available by the authors upon reasonable request.

## References

[1] Rosvall M.D., Fields H.W., Ziuchkovski J., Rosenstiel S.F., Johnston W.M. (2009). Attractiveness, acceptability, and value of orthodontic appliances. Am. J. Orthod. Dentofacial Orthop..

[2] Azaripour A., Weusmann J., Mahmoodi B., Peppas D., Gerhold-Ay A., Van Noorden C.J., Willershausen B. (2015). Braces versus Invisalign^®^: Gingival parameters and patients’ satisfaction during treatment: A cross-sectional study. BMC Oral Health.

[3] Kesling H.D. (1946). Coordinating the predetermined pattern and tooth positioner with conventional treatment. Am. J. Orthod. Oral Surg..

[4] Ponitz R.J. (1971). Invisible retainers. Am. J. Orthod..

[5] Kuo E., Duong T. (2006). Invisalign attachments: Materials. The Invisalign System.

[6] Dasy H., Dasy A., Asatrian G., Rózsa N., Lee H.F., Kwak J.H. (2015). Effects of variable attachment shapes and aligner material on aligner retention. Angle Orthod..

[7] Miller K.B., McGorray S.P., Womack R., Quintero J.C., Perelmuter M., Gibson J., Dolan T.A., Wheeler T.T. (2007). A comparison of treatment impacts between Invisalign aligner and fixed appliance therapy during the first week of treatment. Am. J. Orthod. Dentofacial Orthop..

[8] Karkhanechi M., Chow D., Sipkin J., Sherman D., Boylan R.J., Norman R.G., Craig R.G., Cisneros G.J. (2013). Periodontal status of adult patients treated with fixed buccal appliances and removable aligners over one year of active orthodontic therapy. Angle Orthod..

[9] Kuo E., Miller R.J. (2003). Automated custom-manufacturing technology in orthodontics. Am. J. Orthod. Dentofacial Orthop..

[10] Boyd R.L., Miller R.J., Vlaskalic V. (2000). The Invisalign system in adult orthodontics: Mild crowding and space closure cases. J. Clin. Orthod..

[11] Tomášik J., Zsoldos M., Majdakova K., Fleischmann A., Oravcová Ľ., Sónak Ballová D., Thurzo A. (2024). The potential of AI-powered face enhancement technologies in face-driven orthodontic treatment planning. Appl. Sci..

[12] Bichu Y.M., Alwafi A., Liu X., Andrews J., Ludwig B., Bichu A.Y., Zou B. (2023). Advances in orthodontic clear aligner materials. Bioact. Mater..

[13] Tartaglia G.M., Mapelli A., Maspero C., Santaniello T., Serafin M., Farronato M., Caprioglio A. (2021). Direct 3D printing of clear orthodontic aligners: Current state and future possibilities. Materials.

[14] Hennessy J., Al-Awadhi E.A. (2016). Clear aligners generations and orthodontic tooth movement. J. Orthod..

[15] Gomez J.P., Peña F.M., Martínez V., Giraldo D.C., Cardona C.I. (2015). Initial force systems during bodily tooth movement with plastic aligners and composite attachments: A three-dimensional finite element analysis. Angle Orthod..

[16] Elkholy F., Mikhaiel B., Repky S., Schmidt F., Lapatki B.G. (2019). Effect of different attachment geometries on the mechanical load exerted by PET-G aligners during derotation of mandibular canines: An in vitro study. J. Orofac. Orthop..

[17] Rossini G., Parrini S., Castroflorio T., Deregibus A., Debernardi C.L. (2015). Efficacy of clear aligners in controlling orthodontic tooth movement: A systematic review. Angle Orthod..

[18] Kravitz N.D., Kusnoto B., BeGole E., Obrez A., Agran B. (2009). How well does Invisalign work? A prospective clinical study evaluating the efficacy of tooth movement with Invisalign. Am. J. Orthod. Dentofacial Orthop..

[19] Grünheid T., Loh C., Larson B.E. (2017). How accurate is Invisalign in nonextraction cases? Are predicted tooth positions achieved?. Angle Orthod..

[20] Djeu G., Shelton C., Maganzini A. (2005). Outcome assessment of Invisalign and traditional orthodontic treatment compared with the American Board of Orthodontics objective grading system. Am. J. Orthod. Dentofac. Orthop..

[21] Savignano R., Valentino R., Razionale A.V., Michelotti A., Barone S., D’Antò V. (2019). Biomechanical effects of different auxiliary-aligner designs for the extrusion of an upper central incisor: A finite element analysis. J. Healthc. Eng..

[22] Cai Y., He B., Yang X., Yao J. (2015). Optimization of configuration of attachment in tooth translation with transparent tooth correction by appropriate moment-to-force ratios: Biomechanical analysis. Biomed. Mater. Eng..

[23] Jones M.L., Mah J., O’Toole B.J. (2009). Retention of thermoformed aligners with attachments of various shapes and positions. J. Clin. Orthod..

[24] Al Noor H.S.S., Al-Joubori S.K. (2018). Retention of different orthodontic aligners according to their thickness and the presence of attachments. Int. J. Med. Res. Health Sci..

[25] Jindal P., Worcester F., Siena F.L., Forbes C., Juneja M., Breedon P. (2020). Mechanical behaviour of 3D printed vs thermoformed clear dental aligner materials under non-linear compressive loading using FEM. J. Mech. Behav. Biomed. Mater..

[26] Mantovani E., Castroflorio E., Rossini G., Garino F., Cugliari G., Deregibus A., Castroflorio T. (2018). Scanning electron microscopy evaluation of aligner fit on teeth. Angle Orthod..

[27] Mantovani E., Castroflorio E., Rossini G., Garino F., Cugliari G., Deregibus A., Castroflorio T. (2019). Scanning electron microscopy analysis of aligner fitting on anchorage attachments. J. Orofac. Orthop..

[28] Lombardo L., Palone M., Longo M., Arveda N., Nacucchi M., De Pascalis F., Spedicato G., Siciliani G. (2020). MicroCT X-ray comparison of aligner gap and thickness of six brands of aligners: An in- vitro study. Prog. Orthod..

[29] Cortona A., Rossini G., Parrini S., Deregibus A., Castroflorio T. (2020). Clear aligner orthodontic therapy of rotated mandibular round-shaped teeth: A finite element study. Angle Orthod..

[30] Elkholy F., Schmidt F., Jäger R., Lapatki B.G. (2016). Forces and moments delivered by novel, thinner PET-G aligners during labiopalatal bodily movement of a maxillary central incisor: An in vitro study. Angle Orthod..

[31] Hahn W., Zapf A., Dathe H., Fialka-Fricke J., Fricke-Zech S., Gruber R., Kubein-Meesenburg D., Sadat-Khonsari R. (2010). Torquing an upper central incisor with aligners—Acting forces and biomechanical principles. Eur. J. Orthod..

[32] Faul F., Erdfelder E., Lang A.G., Buchner A. (2007). G*Power 3: A flexible statistical power analysis program for the social, behavioral, and biomedical sciences. Behav. Res. Methods.

[33] Yassen G.H., Platt J.A., Hara A.T. (2011). Bovine teeth as substitute for human teeth in dental research: A review of literature. J. Oral Sci..

[34] de Dios Teruel J., Alcolea A., Hernández A., Ruiz A.J. (2015). Comparison of chemical composition of enamel and dentine in human, bovine, porcine and ovine teeth. Arch. Oral Biol..

[35] Ender A., Mehl A. (2015). In-vitro evaluation of the accuracy of conventional and digital methods of obtaining full-arch dental impressions. Quintessence Int..

[36] Ye N., Wu T., Dong T., Yuan L., Fang B., Xia L. (2019). Precision of 3D-printed splints with different dental model offsets. Am. J. Orthod. Dentofac. Orthop..

[37] Paradowska-Stolarz A., Wezgowiec J., Mikulewicz M. (2023). Comparison of two chosen 3D printing resins designed for orthodontic use: An in vitro study. Materials.

[38] Jindal P., Juneja M., Siena F.L., Bajaj D., Breedon P. (2019). Mechanical and geometric properties of thermoformed and 3D printed clear dental aligners. Am. J. Orthod. Dentofac. Orthop..

[39] Mitić V., Janošević M. (2008). The effect of phosphoric acid application time on the bond strength of orthodontic brackets. Stomatol. Glas. Srb..

[40] Parrish B.C., Katona T.R., Isikbay S.C., Stewart K.T., Kula K.S. (2012). The effects of application time of a self-etching primer and debonding methods on bracket bond strength. Angle Orthod..

[41] Turk T., Elekdag-Turk S., Isci D. (2007). Effects of self-etching primer on shear bond strength of orthodontic brackets at different debond times. Angle Orthod..

[42] Elkholy F., Lapatki B.G. (2018). Recommendation of a novel film-thickness sequence, 0.4, 0.5 and 0.75 mm, for aligner systems. J. Align. Orthod..

[43] Krey K.F., Behyar M., Hartmann M., Corteville F., Ratzmann A. (2019). Behaviour of monolayer and multilayer foils in the aligner thermoforming process. J. Align. Orthod..

[44] Ekşi O., Karabeyoğlu S.S. (2017). The effect of process parameters on thickness distribution in thermoforming. Adv. Sci. Technol. Res. J..

[45] Cowley D.P. (2012). Effect of Gingival Margin Design on Retention of Thermoformed Orthodontic Aligners. Master’s Thesis.

[46] Apicella D., Veltri M., Chieffi N., Polimeni A., Giovannetti A., Ferrari M. (2011). Implant adaptation of stock abutments versus CAD/CAM abutments: A radiographic and scanning electron microscopy study. Ann. Stomatol..

[47] Wagner R.J. (2000). Techniques in Biological Electron Microscope.

[48] Bozzola J.J., Russell L.D. (1999). Electron Microscopy: Principles and Techniques for Biologists.

[49] Son K., Lee S., Kang S.H., Park J., Lee K.B., Jeon M., Yun B.J. (2019). A comparison study of marginal and internal fit assessment methods for fixed dental prostheses. J. Clin. Med..

[50] Ryokawa H., Miyazaki Y., Fujishima A., Miyazaki T., Maki K. (2006). The mechanical properties of dental thermoplastic materials in a simulated intraoral environment. Orthod. Waves.

[51] Bucci R., Rongo R., Levatè C., Michelotti A., Barone S., Razionale A.V., D’Antò V. (2019). Thickness of orthodontic clear aligners after thermoforming and after 10 days of intraoral exposure: A prospective clinical study. Prog. Orthod..

[52] Burashed H. (2023). Quantifying the efficacy of overbite reduction in patients treated with clear aligners using optimized versus conventional attachments. J. World Fed. Orthod..

[53] Li Q., Zhang F., Xu X., Chen J. (2025). Impacts of surface wear of attachments on maxillary canine distalization with clear aligners: A three-dimensional finite element study. Front. Bioeng. Biotechnol..

[54] Hassanaly T., Smith R., Johnson M. (2024). A comparison of the upper anterior teeth movements with optimized and conventional attachment. J. Clin. Exp. Dent..

[55] Groody J.T., Miller K., Williams P. (2023). Effect of clear aligner attachment design on extrusion of maxillary lateral incisors: A multicenter, single-blind randomized clinical trial. Am. J. Orthod. Dentofac. Orthop..

[56] Simon M., Keilig L., Schwarze J., Jung B.A., Bourauel C. (2014). Forces and moments generated by removable thermoplastic aligners: Incisor torque, premolar derotation, and molar distalization. Am. J. Orthod. Dentofac. Orthop..

[57] Barone S., Paoli A., Neri P., Razionale A.V., Giannese M., Eynard B., Nigrelli V., Oliveri S.M., Peris-Fajarnes G., Rizzuti S. (2017). Mechanical and geometrical properties assessment of thermoplastic materials for biomedical application. Advances on Mechanics, Design Engineering and Manufacturing.

[58] Ryu J.H., Kwon J.S., Jiang H.B., Cha J.Y., Kim K.M. (2018). Effects of thermoforming on the physical and mechanical properties of thermoplastic materials for transparent orthodontic aligners. Korean J. Orthod..

[59] Lombardo L., Martines E., Mazzanti V., Arreghini A., Mollica F., Siciliani G. (2017). Stress relaxation properties of four orthodontic aligner materials: A 24-hour in vitro study. Angle Orthod..

[60] Robertson L., Kaur H., Fagundes N.C.F., Romanyk D., Major P., Flores Mir C. (2020). Effectiveness of clear aligner therapy for orthodontic treatment: A systematic review. Orthod. Craniofac. Res..

[61] Gao L., Tian Y., Liu W., Song J. (2024). Artificial intelligence in orthodontics: A systematic review and meta-analysis of diagnostic performance and treatment prediction. Am. J. Orthod. Dentofac. Orthop..

